# Performance of Sustainable Fly Ash and Slag Cement Mortars Exposed to Simulated and Real In Situ Mediterranean Conditions along 90 Warm Season Days

**DOI:** 10.3390/ma10111254

**Published:** 2017-10-31

**Authors:** José Marcos Ortega, María Dolores Esteban, Isidro Sánchez, Miguel Ángel Climent

**Affiliations:** 1 Departamento de Ingeniería Civil, Universidad de Alicante, Ap. Correos 99, 03080 Alacant/Alicante, Spain; isidro.sanchez@ua.es (I.S.); ma.climent@ua.es (M.Á.C.); 2 Departamento de Ingeniería Civil, Urbanismo y Aeroespacial, Escuela de Arquitectura, Ingeniería y Diseño, Universidad Europea, c/Tajo s/n, 28670 Villaviciosa de Odón, Madrid, Spain; mariadolores.esteban@universidadeuropea.es

**Keywords:** ground granulated blast-furnace slag, fly ash, sustainability, real condition exposure, non-optimum laboratory condition, Mediterranean climate environment, temperature, relative humidity, durability, ordinary Portland cement

## Abstract

Nowadays, cement manufacture is one of the most polluting worldwide industrial sectors. In order to reduce its CO_2_ emissions, the clinker replacement by ground granulated blast–furnace slag and fly ash is becoming increasingly common. Both additions are well-studied when the hardening conditions of cementitious materials are optimum. Therefore, the main objective of this research was to study the short-term effects of exposure, to both laboratory simulated and real in situ Mediterranean climate environments, on the microstructure and durability-related properties of mortars made using commercial slag and fly ash cements, as well as ordinary Portland cement. The real in situ condition consisted of placing the samples at approximately 100 m away from the Mediterranean Sea. The microstructure was analysed using mercury intrusion porosimetry. The effective porosity, the capillary suction coefficient and the non-steady state chloride migration coefficient were also studied. In view of the results obtained, the non-optimum laboratory simulated Mediterranean environment was a good approach to the real in situ one. Finally, mortars prepared using sustainable cements with slag and fly ash exposed to both Mediterranean climate environments, showed adequate service properties in the short-term (90 days), similar to or even better than those in mortars made with ordinary Portland cement.

## 1. Introduction

At present, cement manufacture is one of the most pollution-producing industrial sectors worldwide. Improving the sustainability of cement industry is still an important challenge and is mainly focused on lessening the CO_2_ emissions produced by cement production. In this way, the use of additions as clinker replacement is becoming increasingly common [1,2,3,4,5,6,7,8,9]. Furthermore, many of those supplementary cementitious materials are pollutant wastes produced along other industrial processes, so their reuse would partly solve other environmental problems, such as their storage.

Two of the most popular additions are ground granulated blast-furnace slag and fly ash, and several researches [10,11,12,13] have noted that cementitious materials, which incorporate these additions, have a better performance compared to those made only with ordinary Portland cement (OPC). This good behaviour is related to the slag hydration and fly ash pozzolanic reactions, whose products are additional CSH phases, which produce a more refined pore network [6,7,10,14], as well as an improvement of the service properties of cement-based materials [12,15,16,17,18,19].

Nevertheless, in the majority of those studies [10,11,17,20,21], the materials were exposed to optimum laboratory conditions, while real structures are hardened in different environments depending on their geographical location. In that regard, there are several studies [15,16,18,19,22,23,24,25] in which the behaviour of slag and fly ash cement-based materials exposed to real in situ conditions has been analysed. However, the high variability of temperature and relative humidity, as well as other weather parameters, along the exposure period of materials to real environments make it difficult to analyse the specific influence of each one of them, producing uncertainty. For that reason, and as an approach to those real hardening environmental conditions, several studies [4,21,26,27,28,29,30,31,32] have focused on cementitious materials with slag and fly ash that were kept under non-optimum laboratory hardening conditions, which combined constant temperature and relative humidity (RH), different to the optimum values of 20 °C and 100% RH.

In general, the performance of slag and fly ash mortars and concretes hardened under non-optimum laboratory conditions was adequate [4,27,28,33], mainly when the values of temperature and relative humidity are high [21,27,28,34]. On the other hand, in view of the results of the abovementioned researches [15,16,18,22,23,24,25,35,36], in which slag and fly ash cement-based materials were exposed to real in situ environments, it seems that their behaviour changes based on the climate conditions of the site where the specimens were located. In addition to this, there are no experimental studies in which the performance of slag and fly ash cementitious materials hardened in both real and simulated conditions of Mediterranean climate was compared.

Therefore, the main objective of this research is to study the short-term effects of the exposure to both laboratory simulated and real in situ Mediterranean climate environments on the microstructure and durability-related properties of mortars made using commercial ground granulated blast-furnace slag and fly ash cements. The real in situ condition consisted of placing the samples at approximately 100 m from the Mediterranean Sea, for three months during the warm season, so they were exposed to the action of airborne chlorides. The performance of fly ash and slag mortars was compared to that noted for ordinary Portland cement (OPC) ones. Moreover, samples of all studied cement types were also kept under an optimum laboratory condition, in order to get a reference of behaviour for analysing the influence of non-optimum environments. With respect to the experimental techniques used in this research, the microstructure has been analysed using mercury intrusion porosimetry. Furthermore, the studied durability-related parameters were effective porosity, the capillary suction coefficient and the non-steady state chloride migration coefficient.

## 2. Materials and Methods

### 2.1. Sample Preparation

All tests were performed on mortars. They were made with three commercial cements, an ordinary Portland cement (OPC), CEM I 42.5 R (CEM I from now on), a ground granulated blast-furnace slag cement (with a content of slag from 66% to 80% of total binder), III/B 42.5 L/SR (CEM III for now on) and a fly ash pozzolanic cement (content of fly ash between 36% and 55%), CEM IV/B(V) 32.5 N (CEM IV hereafter), according to the standard UNE-EN 197-1 [37]. The different components of each one of the commercial cements and their percentage of the total binder are detailed in Table 1. The specimens were prepared with a water-to-cement ratio of 0.5. Fine aggregate was used according to the standard UNE-EN 196-1 [38], and the aggregate-to-cement ratio was 3:1 for all the mortars.

The prepared specimens were cylinders, which were cast in moulds of 10 cm diameter and 15 cm height. For the first 24 h, they were kept in 95% RH chamber and 20 °C. After that time, they were de-moulded and were cut to obtain slices of approximately 1 cm thickness and cylinders of 5 cm thickness. Finally, the tests were performed at 28 and 90 hardening days.

### 2.2. Environmental Conditions

Three different environmental exposure conditions were studied (see Table 2). Firstly, an optimum laboratory condition, with 20 °C and 100% relative humidity (RH), called environment A, was studied as a reference to compare the influence of the rest of the environmental conditions in the microstructure and durability-related properties of the mortars. The second environment was a non-optimum laboratory environment representative of the Mediterranean climate, present in the eastern part of the Iberian Peninsula (Spain and Portugal), and it was called environment B. The mortars hardened under this environment B were exposed to a constant 20 °C temperature and 65% RH, which correspond to the annual average values of both parameters for the Mediterranean climate.

In order to simulate the conditions (temperature and RH) of the abovementioned environments (A and B), the mortar samples were introduced into hermetically sealed recipients containing water or glycerol solutions, and these containers were placed into different chambers with controlled temperature. The appropriate concentration of the glycerol solutions were selected in order to achieve the target relative humidity value, according to the standard DIN 50 008 part 1 [39]. The mortar samples were stored into the containers, without contact with the solutions.

**Figure 1:**
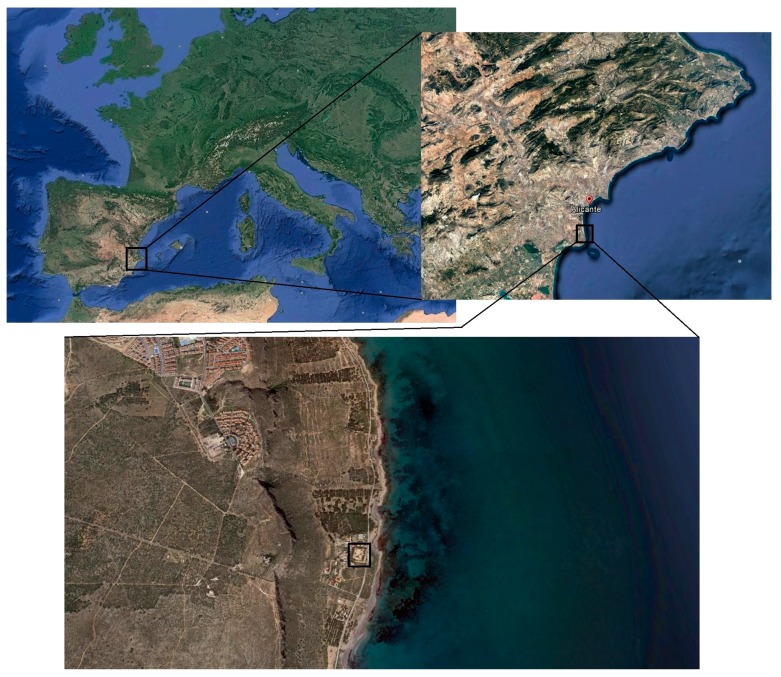
Location of the real exposure site (black line squares) for environment C. The samples were placed in an exposure station located in the Santa Pola’s Cape (Alicante province, Spain). The distance from this exposure site to the Mediterranean Sea was about 100 m. The satellite images were obtained using the Google Earth software (Google, Santa Clara, CA, USA).

**Figure 2:**
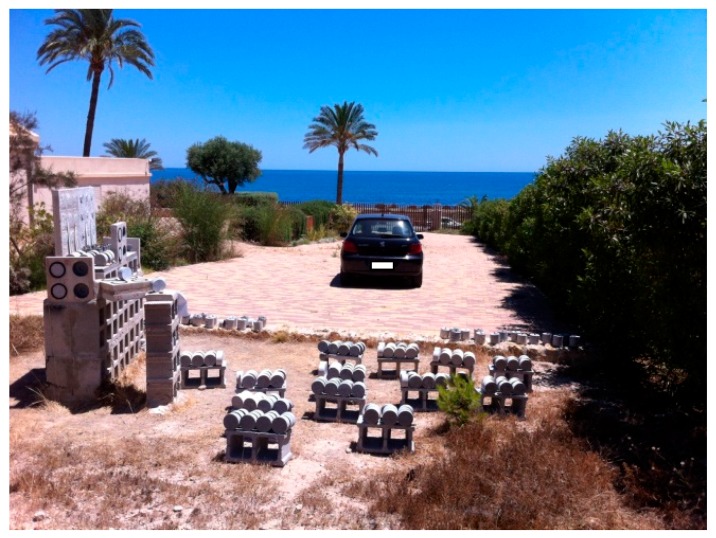
Image of the samples placed in the exposure station of Santa Pola’s Cape (province of Alicante). As can be observed, the distance from the Mediterranean Sea is about 100 m.

The third environment consisted of exposing the samples to a maritime real condition of Mediterranean climate, and it was called environment C. The samples were placed in an exposure station, located in the Santa Pola’s Cape (Alicante province, Spain) (see Figure 1), at approximately 100 m from the Mediterranean Sea, as can be observed in Figure 2. Therefore, the samples exposed to this environment C would be affected by the action of the airborne chlorides coming from sea water. In view of that, the location of the samples would accomplish the specifications of exposure class IIIa defined by the Spanish Code on Structural Concrete EHE-08 [40], which would be equivalent to exposure class XS1 (corrosion induced by chlorides from sea water without direct contact with it) indicated by the Eurocode 2 [41]. The group of samples exposed to environment C, were cured in a chamber at 95% RH and 20 °C along their first seven hardening days, before being placed in the exposure site. Many studies have shown [16,18,28,36] the importance of curing in the development of service properties of cementitious materials exposed to real environments. For this reason, in this work it was selected a curing period of seven days.

**Figure 3:**
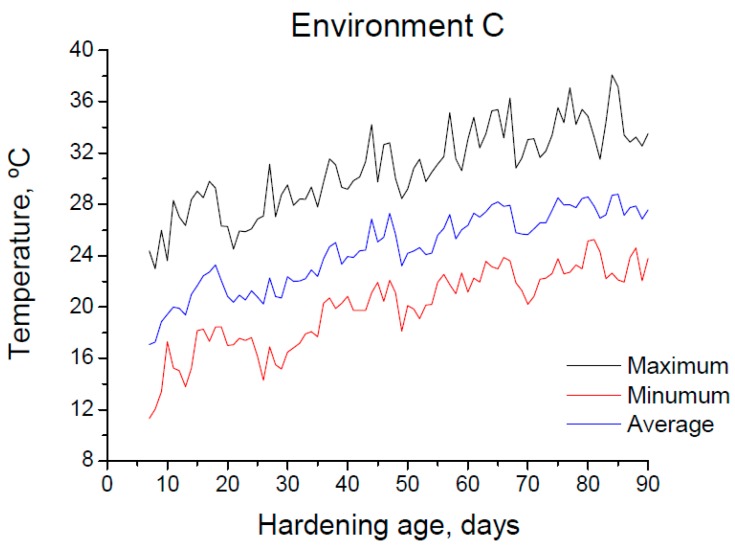
Daily maximum, minimum and average temperature registered in the station throughout the 90-day period of exposure to environment C.

**Figure 4:**
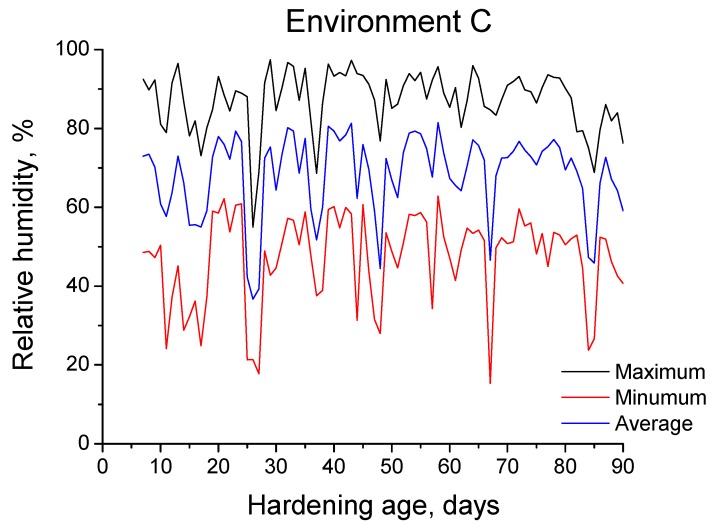
Daily maximum, minimum and average relative humidity registered in the station over the 90-day period of exposure to environment C.

**Figure 5:**
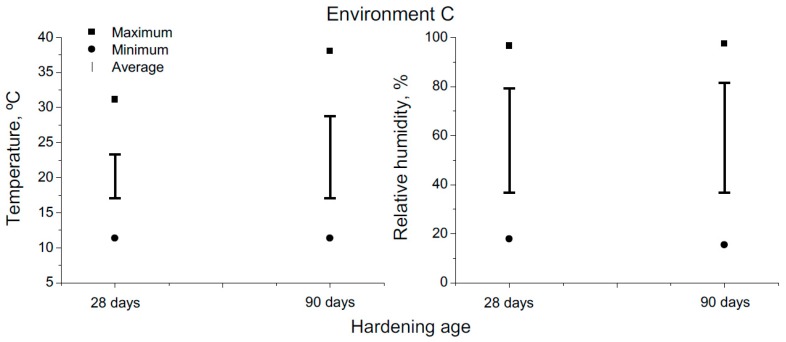
Absolute maximum, absolute minimum and interval of average temperature and relative humidity at which the samples were exposed to environment C for 28 days- and 90 days-period.

The 90-days period of exposure to environment C covered the months from May to July, which belong to the warm season in the site. The evolution of daily maximum, minimum and average temperatures and RH registered in the station throughout the 90-days exposure interval are shown in Figure 3 and Figure 4, respectively. Furthermore, Figure 5 shows the absolute maximum, the absolute minimum and the interval of average environmental temperature and relative humidity at which the samples were exposed over both 28-days and 90-days periods. As has been noted, both temperature and RH showed high variability. Regarding the temperature, its average value for specimens tested at 90 days was approximately 23 °C, although they were exposed to a range of temperatures between 10 °C and 38 °C. On the other hand, the interval of RH at which those samples were subjected covered the values from 15 to 98% (average value 59%).

**Figure 6:**
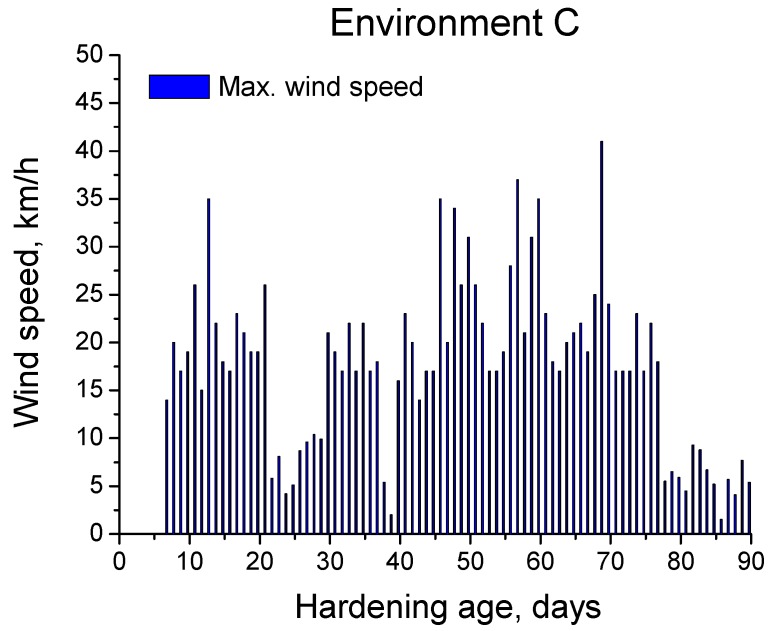
Daily maximum wind speed registered in the station over the exposure period to environment C.

Finally, rain and wind are other environmental factors which could influence the water saturation degree of the samples, and as a consequence, the development of their microstructure and service properties. With respect to rain, it did not rain over the studied period. Wind is another factor which could affect the hydration of the samples, because fast wind could accelerate their drying process. The daily maximum wind speed registered in the station during the exposure interval is depicted in Figure 6. As can be observed, the maximum wind speed also showed high variability and its average value was about 20 km/h, although several peaks greater than 35 km/h were observed.

### 2.3. Mercury Intrusion Porosimetry

Mercury intrusion porosimetry (MIP) was used for studying the microstructure of the hardened mortar samples. This is a well-known and extensively used technique, in spite of their main reported problems [6,7,42,43]. Prior to the test, samples were oven-dried for 24 h at 105 °C. The porosimeter used was a MicromeriticsAutopore IV 9500 (Norcross, GA, USA), which allows pore diameter determination in the range from 0.9 mm to 5 nm. Duplicate MIP measurements were performed on each material and for each condition and age. The tested samples were obtained from slices with approximately 1 cm thickness. Total porosity and pore size distribution were studied. The pore size distribution was analysed with the curves logarithm of differential intrusion volume versus pore size, as well as representing the relative volume of pores of the following fixed diameter ranges: <10 nm, 10–100 nm, 100 nm–1 µm, 1–10 µm, 10 µm–0.1 mm and >0.1 mm [7,17].

### 2.4. Capillary Absorption Test

The capillary absorption test was performed according to the standard UNE 83982 [44]. This test is based on the Fagerlund method to obtain the capillarity of concrete. Before the test, cylindrical samples of 10 cm diameter and 5 cm thickness were subjected to a pre-conditioning procedure, which firstly consisted of a complete drying in an oven at 105 °C for 12 h, and from then to the start of the test they were saved in a hermetically sealed recipient with silica gel during the next 12 h [17,34]. The reason for using this pre-conditioning procedure, instead of longer ones [45], was to avoid the contact of the samples with water during a long time period, which could modify the hydration degree of the material, influencing the consequent development of their microstructure and properties.

Prior to the test, the lateral surface of the sample was sealed using self-adhesive tape [45]. After that, the samples were introduced into a container with a flat base [44], which was filled with distilled water up to 5±1 mm on the lateral surface [44], resulting in more than a 95% of the base of the sample being in contact with water. Throughout the test, water level was kept constant and the container was hermetically closed. Samples were weighed at different times set in the standard [44]. The test finished when the difference between two consecutive weights, with 24 h difference, was lower than 0.1%, in mass. The capillary suction coefficient and effective porosity were calculated according to the expressions:

(1) $$\varepsilon_{e}\;=\;\frac{Q_{n}\;-\;Q_{0}}{A\cdot h\cdot \delta_{a}}$$

(2) $$K\;=\;\frac{\delta_{a}\cdot \varepsilon_{e}}{10\cdot \sqrt{m}}\;\mathrm{with}\;m\;=\;\frac{t_{n}}{h^{2}}$$

where: ε_e_ is the effective porosity, Q_n_ is the weight of the sample at the end of the test (g), Q_0_ is the weight of the sample before starting the test (g), A is the surface of the sample in contact with water (cm^2^), h is the thickness of the sample (cm), δ_a_ is the density of water (1 g/cm^3^), K is the capillary suction coefficient (kg/m^2^min^0.5^), m is the resistance to water penetration by capillary suction (min/cm^2^) and t_n_ is the time necessary to reach the saturation (minutes).

For each cement type and condition, three different specimens were tested at each age.

### 2.5. Forced Migration Test

The forced chloride migration test was performed according to NT Build 492 [46]. The result of this test is the non-steady-state chloride migration coefficient D_NTB_. For each cement type and environment, three different cylindrical specimens of 10 cm diameter and 5 cm height were tested.

## 3. Results

### 3.1. Mercury Intrusion Porosimetry

**Figure 7:**
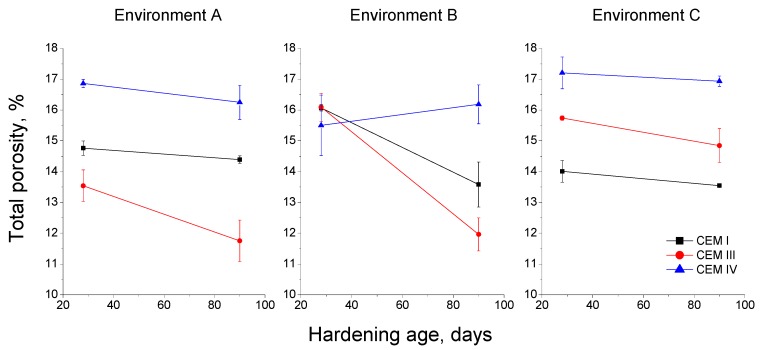
Results of total porosity for the studied mortars.

The results of total porosity as a function of hardening time in days are shown in Figure 7 for each environment. For environment A, the total porosity of CEM III mortars decreased between 28 and 90 hardening days, and it showed the lowest values of all the studied samples. This porosity also decreased for CEM I and CEM IV specimens exposed to environment A, although their values were higher than those observed for the CEM III ones. In relation to environment B, at 28 days the total porosity was very similar for all the mortars. Nevertheless, at 90 days this parameter hardly changed for CEM IV specimens, and it fell for the CEM I and III ones, being slightly lower for those mortars made using slag cement. For environment C, the total porosity hardly decreased with time for all the specimens. The highest values of this parameter for this condition were noted for CEM IV mortars and the lowest for the CEM I ones.

**Figure 8:**
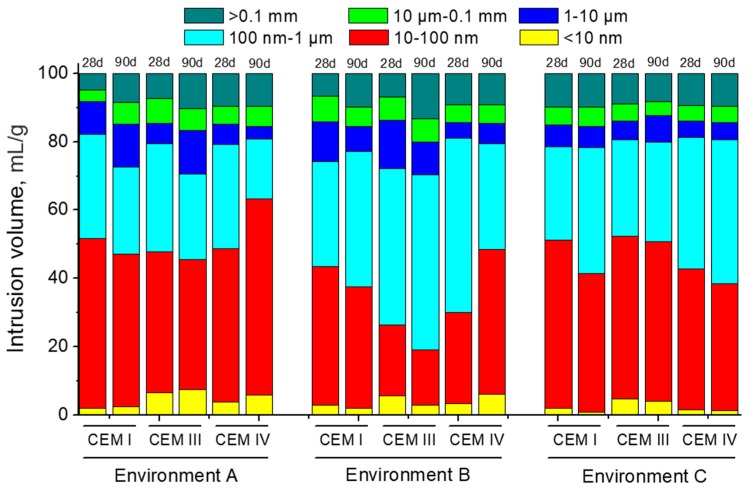
Pore size distributions for CEM I, III and IV mortars obtained for each environment.

The pore size distributions for CEM I, III and IV mortars are depicted in Figure 8. For environment A, it is important to emphasize that microstructure was more refined in the long-term for mortars prepared using cements with additions, as shown by their higher proportion of smaller pores (sizes less than 10 nm) compared to CEM I mortars. The porous network hardly became more refined from 28 to 90 days for CEM I and III specimens hardened under environment A. However, for CEM IV mortars, there was an important pore refinement from 28 to 90 days, as shown by the rise of percentage of pores with diameters in the range of 10–100 nm. Regarding environment B, the less refined pore network corresponded to CEM III mortars. Furthermore, a loss of pore refinement with age for CEM I and III specimens was observed for this condition, while this microstructure refinement increased for CEM IV mortars between 28 and 90 days. For environment C, mortars prepared with CEM IV showed the least refined pore structure, whereas the most refined one was noted for CEM III specimens. In addition to this, a loss of pore refinement with time for CEM I and IV mortars exposed to environment C was noted, while the microstructure of CEM III ones hardly changed.

**Figure 9:**
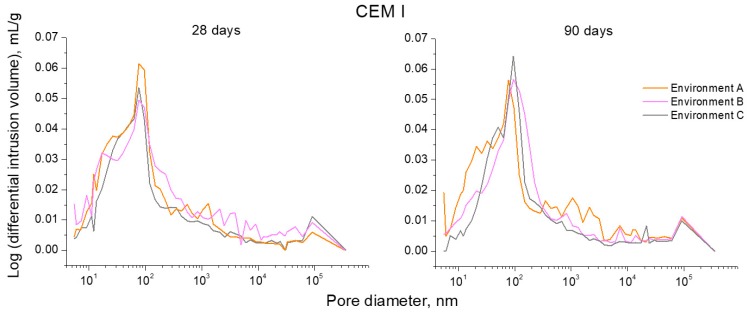
Curves logarithm of differential intrusion volume versus pore size obtained at 28 and 90 hardening days for CEM I mortars exposed to the three studied environments.

**Figure 10:**
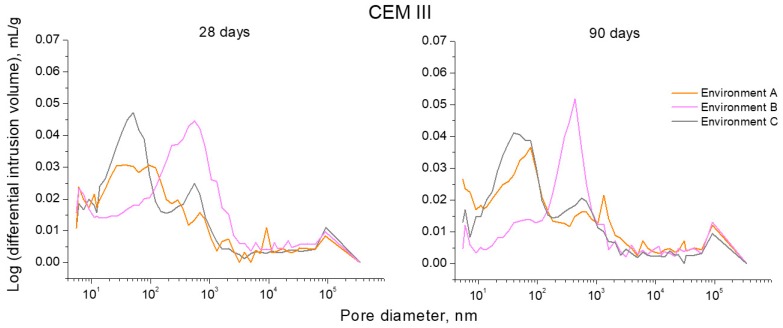
Curves logarithm of differential intrusion volume versus pore size obtained at 28 and 90 hardening days for CEM III mortars exposed to the three studied environments.

**Figure 11:**
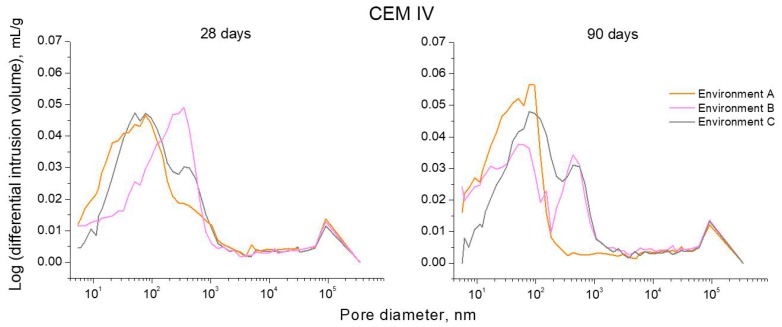
Curves logarithm of differential intrusion volume versus pore size obtained at 28 and 90 hardening days for CEM IV mortars exposed to the three studied environments.

The logarithm curves of differential intrusion volume versus pore size obtained for CEM I, III and IV mortars can be observed in Figure 9, Figure 10 and Figure 11, respectively. For CEM I mortars, the main family of pores at 28 and 90 days was about a diameter of 100 nm, independently of the environmental condition, as would indicate the highest peak of curves depicted in Figure 9. On the other hand, for CEM III mortars (see Figure 10), along the studied time period, the main pore family was noted between 10 and 100 nm for environment A, while for environment B this family belonged to the pore size range between 100 nm and 1 µm. Furthermore, for slag mortars exposed to environment C, two families have clearly been noted, the main one showed a size less than 100 nm and the other family was close to 1 µm diameter. For CEM IV mortars (see Figure 11) exposed to environment A, the main family of pores was around 100 nm of diameter. For environment B, fly ash mortars showed one pore family near 1 µm of size at 28 days, whereas at 90 days, two similar families have been observed for these samples close to 100 nm and 1 µm, respectively. Lastly, there also were two noticeable families for CEM IV specimens kept in environment C, and again the highest peak was around 100 nm and the other was about 1 µm.

### 3.2. Capillary Absorption Test

**Figure 12:**
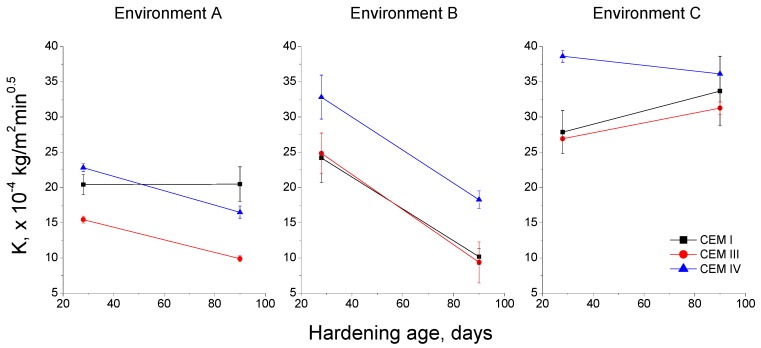
Capillary suction coefficient (K) results for each environment.

The capillary suction coefficient K and the effective porosity of the samples were obtained. The results of the capillary suction coefficient K are depicted in Figure 12. For environment A, the lowest values of this coefficient were noted for CEM III mortars, while it was similar for CEM I and IV mortars. The studied mortars kept under environment B showed higher coefficients K at 28 days compared to environment A. Nevertheless, this parameter fell with time for all of them, and at 90 days it was, overall, similar to environment A. For environment C, it has been noted the highest coefficient K values for all the cement types, and this parameter hardly changed along the studied period, or even it increased, as happened for CEM I and III specimens.

**Figure 13:**
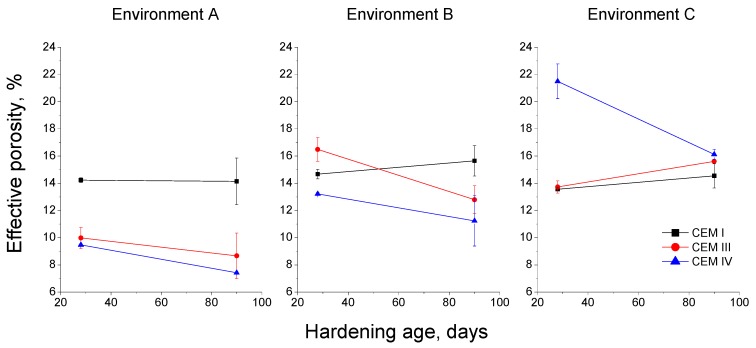
Results of effective porosity obtained for concretes the studied mortars.

The results of effective porosity are shown in Figure 13. This parameter slightly changed along the studied period for environment A, with its values for CEM III and IV mortars being lower than CEM I ones. For environment B, in general, the effective porosity was higher compared to environment A, and at 90 days fly ash and slag cement mortars showed lower values of this parameter than those prepared using CEM I. For environment C, effective porosity was practically constant, and it was similar for CEM I and III mortars, although for CEM IV this parameter was relatively high at 28 days, decreasing from then to 90 days, and it showed at that age a similar value to the rest of studied mortars.

### 3.3. Forced Migration Test

**Figure 14:**
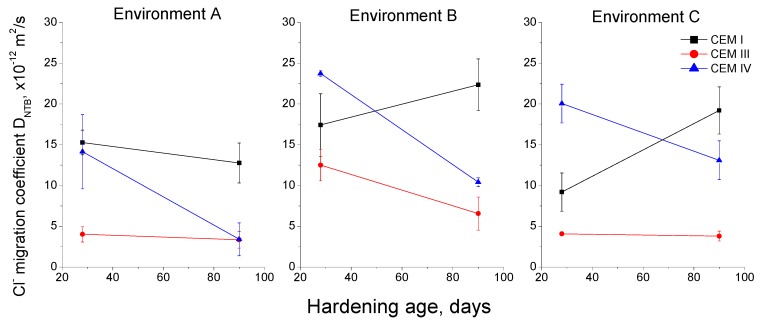
Results of non-steady-state chloride migration coefficient for the studied mortars.

The results of the non-steady-state chloride migration coefficient D_NTB_ for each environment can be observed in Figure 14. For environment A, this coefficient decreased with age for all the studied mortars, especially for the CEM IV ones, and at 90 days its values were lower for mortars with active additions than for those made with CEM I. For environment B, the migration coefficient fell with time for CEM III and IV mortars, and it increased for the CEM I ones. Again, slag and fly ash mortars exposed to environment B showed lower values of this coefficient at 90 days compared to those prepared using CEM I. For environment C, migration coefficient decreased for CEM IV specimens, increased for CEM I ones and stayed practically constant for those made with CEM III. For this environment, at 90 days, mortars with active additions also showed lower migration coefficients than CEM I ones.

## 4. Discussion

### 4.1. Microstructure Characterisation

In relation to the total porosity results (see Figure 7), the decreasing overall tendency noted for the studied mortars exposed to environment A could be related to the development of clinker and slag hydration, and fly ash pozzolanic reactions, which would form new solid phases [4,10], reducing the total porosity. The highest values of this parameter observed for CEM IV mortars kept under environment A and, in general under the other environments, would be in keeping with the results obtained by other authors [10,11], who have pointed out that the addition of fly ash would not entail a porosity reduction, although it would produce a higher pore network refinement. Moreover, the delay of fly ash pozzolanic reactions compared to clinker and slag hydration [47,48] could have also influenced this relatively high porosity values along the relatively short ages studied. The lowest total porosity showed for CEM III mortars exposed to environment A, would also agree with the findings of other authors [4,10,27], who have indicated the beneficial effect of slag hydration in the microstructure of cement-based materials.

The specimens prepared using CEM I and CEM III showed higher total porosity values at 28 days for environment B compared to environment A. This result could be related to the lower relative humidity (65%) of the non-optimum condition B. High availability of water in the environment would favour the development of clinker and slag hydration [4,28]. Therefore, the lower relative humidity of this environment would slow down those hydration reactions [4,28,30,31], and more time would be needed to observe a porosity reduction, as suggested by the relative low values of this parameter noted for CEM I and III mortars at 90 days. The total porosity of CEM IV specimens hardened in environment B hardly changed with age, but their values did not greatly differ in comparison to environment A, so it seems that the lower relative humidity would scarcely affect the porosity of these fly ash mortars. For environment C, the lowest total porosities corresponded to CEM I mortars and their values were very similar to those noted for the same type of mortar exposed to environment A. Then, the real in situ environment would not have a great influence in the total porosity of mortars without additions. The slag and fly cement mortars generally showed higher total porosities for environment C compared to the rest of the studied ones. This could be explained by the changeable temperature and relative humidity of this real condition, which would influence the development of slag hydration and fly ash pozzolanic reactions [4,27,28,30,31,33,36].

Regarding pore size distributions (see Figure 8), the highest pore refinement observed for all the mortars exposed to environment A, compared to the rest of studied conditions, could be due to the optimum temperature and relative humidity of this environment, which would facilitate the development of clinker and slag hydration [4,10], as well as the fly ash pozzolanic reactions [28,33]. As products of these reactions, solid phases would be made, which would reduce the pore sizes [10,49,50]. The higher pore refinement showed by slag and fly cement mortars would coincide with the results of other authors [10,15,50], and it would be explained as a consequence of additional CSH phases produced by the slag hydration and fly ash pozzolanic reactions, compared to clinker hydration [10,47,48].

For CEM I mortars, a slight loss of pore refinement has been observed for environments B and C in comparison to condition A, as suggested by the reduction of volume of pores with diameters lower than 100 nm (see Figure 8), as well as the scarce variation of the main peak of their logarithm curves of differential intrusion volume versus pore size (see Figure 9), especially at 90 days. Comparing the pore size distribution for CEM I mortars among environments B and C, a higher microstructure refinement for condition C has been noted. This could be due to the higher maximum daily temperatures and relative humidities to which the samples were exposed in this real in situ environment (see Figure 6). Despite their variability, the high values of these parameters along specific moments of each exposure day, could favour the development of clinker hydration [4,27,28], and its resulting solid formation.

A similar result has been observed for CEM III specimens exposed to environments B and C. However, for those samples prepared using slag cement, the difference among both conditions was more pronounced (see Figure 8). For environment B, CEM III mortars showed an important loss of pore refinement compared to the optimum condition A, which was corroborated by the fact that the main pore family shifted towards higher diameters, as would indicate by their logarithm curves of differential intrusion volume versus pore size (see Figure 10). This could be due to the relatively low relative humidity of environment B, which would notably affect the development of slag hydration [4], making new solid formations more difficult. On the other hand, the pore network of CEM III samples hardened in environment C were hardly less refined than those noted for environment A. As has been explained for CEM I mortars, the higher temperature and relative humidity values reached in several periods of each exposure day to real in situ environment would facilitate the development of slag hydration. In that regard, several studies [4,27] have pointed out that the hardening temperature has an important influence in the development of microstructure and properties of slag cement based-materials. Therefore, the effect of high maximum temperature values for environment C (see Figure 6) had possibly played an important role in the relatively good results related to slag mortar microstructure obtained here.

Pore size distributions obtained for CEM IV mortars exposed to non-optimum conditions were less refined compared to those observed for environment A. At 28 days, the pore refinement of fly ash mortars was greater for those hardened in environment C than for condition B ones, as suggested by their higher percentage of pores with diameters less than 100 nm (see Figure 8), as well as the lower size of their main pore family (see Figure 11). As happened for CEM I and III mortars, this result could be due to the high temperature and relative humidity values achieved along part of each exposure day, which would accelerate the clinker hydration [4,27,28], allowing a faster beginning of fly ash pozzolanic reactions [33,47,48]. As a consequence, their effects were noted at early stages. Nevertheless, at 90 days, the pore size distribution was scarcely more refined for environment B, but not much different in comparison with the C environment. The important pore refinement produced from 28 to 90 days for CEM IV samples exposed to environment B, could be related to the slowing down produced by the lower constant relative humidity of this condition in the development of clinker hydration [4]. The effects of fly ash pozzolanic reactions in the pore network would be more delayed [33,34] and it would be more noticeable at greater ages, as is indicated by the results obtained.

### 4.2. Durability-Related Parameters

Firstly, the capillary suction coefficient K gives information about water ingress in the materials. The study of this parameter is important because water is the mean medium by which the ingress of aggressive ions is produced. For environment A, the values of the coefficient K (see Figure 12) were relatively low at 28 and 90 days, compared to the rest of the environments. These results would coincide with those obtained during the microstructure characterisation, and they would show the effects of a condition with both optimum temperature and relative humidity, which would favour the development of clinker and slag hydration [4,28], as well as fly ash pozzolanic reactions [28,29,33], reducing the coefficient K. For environment B, this parameter showed relatively high values at 28 days, but it greatly fell at 90 days. Again, this result was in keeping with previously discussed microstructure parameters. As has been explained, the great initial values of coefficient K would be produced by the relatively low relative humidity in the environment, which would slow down the development of hydration and pozzolanic reactions [4,28,51,52]. Furthermore, the highest values of this coefficient for environment B were noted for CEM IV mortars, which would indicate the delay of pozzolanic reactions of fly ash compared to clinker and slag hydration [33,47,48]. This is more noticeable for this environment B due to the slowing down of this hydration. For environment C, the coefficient K hardly changed with time and it showed the highest values of the all the studied conditions. Therefore, the beneficial effects of this real in situ condition on the microstructure for some of the analysed mortars, were not been noted for coefficient K. This could be due to the fact that the global percentage of capillary pores was similar, or even higher, for environment C, in spite of having a slightly greater pore refinement.

The effective porosity provides data about the fraction of the material accessible to water and, therefore for aggressive substances, such as chlorides [53]. The effective porosity results (see Figure 13) obtained for environment A were very similar to those obtained for coefficient K, as already discussed. In addition to this, it is important to note the lower effective porosity showed by slag and fly ash cement mortars exposed to this condition in comparison with CEM I ones, which would agree with their higher pore network refinement. Regarding environment B, the decrease of this porosity with time was not as noticeable as observed for coefficient K, although their higher values would denote the abovementioned effect of lower relative humidity of this condition [4,28]. Lastly, for environment C, the effective porosity hardly changed for CEM I and III mortars, and it fell for the CEM IV ones, despite the fact that at 90 days, the values of this parameter were similar to those obtained for environment B. As happened with coefficient K, the improvement of microstructure noted for some of the studied mortars kept under this environment C seemed not to have an influence on the effective porosity. The decrease of this parameter in the medium-term for CEM IV mortars would indicate the effects of the fly ash pozzolanic reactions, and the delay compared to clinker and slag hydration [33,47,48]. Nevertheless, it is interesting to emphasize that for all environments, both effective porosity and coefficient K were overall similar, or even lower, for CEM III and IV mortars at 90 days in comparison to those made with CEM I.

The characterisation of chloride ingress resistance is important, because the corrosion of reinforcing elements embedded in cement-based materials is mainly produced by this aggressive ion. Here, the resistance of the studied mortars against chloride ingress has been studied with the non-steady-state chloride migration coefficient D_NTB_. In general, the result of this coefficient showed similarities to the rest of the studied parameters in this work. The lowest values of the migration coefficient were noted for environment A, which would coincide with previously discussed results. For all the environments, the initial values of this parameter were relatively high for CEM IV mortars, which again would show the delay of fly ash pozzolanic reactions compared to clinker and slag hydration [33,47,48]. On the other hand, the initial values of the migration coefficient were higher for environment B, which would be in agreement with the described parameters, and explained by its lower constant relative humidity. In relation to environment C, it had no deleterious effects in this coefficient, and was even beneficial for chloride ingress resistance of CEM III mortars, which would coincide with microstructure characterisation. Lastly, at 90 days, fly ash and slag mortars showed lower migration coefficients for all environments in comparison to CEM I ones. This would be in keeping with other researches [15,16,17,21], who have pointed out that the addition of fly ash and slag improves the chloride ingress resistance of cement-based materials. On one hand, the greater pore network refinement produced by slag and fly ash in comparison to pure clinker [10,14,17] could contribute to the improvement of this property. On the other hand, the good migration coefficients obtained for CEM III and IV mortars exposed to environments B and C, in which their pore refinement was lower, could be influenced by the higher binding capacity of fly ash and slag cements compared to ordinary Portland cement. This binding capacity is due to the high content of calcium aluminates brought by the fly ash and slag [54]. These aluminates, formed as a result of the hydration of the high Al_2_O_3_ content in blast furnace slag and fly ash, react with chlorides to form chloraluminates, thereby preventing ion diffusion across the material [54].

## 5. Conclusions

The main conclusions that can be drawn from the discussed results can be summarized as follows:

- For all the studied environments, the pore network of slag and fly ash cement mortars was overall more refined at 90 days than that noted for CEM I ones. This result would be explained based on the additional solid phases produced by slag hydration and fly ash pozzolanic reactions.
- The delay in the beginning of fly ash pozzolanic reactions compared to clinker and slag hydration was more noticeable for both laboratory simulated and real in situ Mediterranean climate environments than for optimum condition. This would be due to the slowing down of portlandite formation as a product of clinker hydration in non-optimum environments, due to the lower availability of water.
- The relatively high temperature and relative humidity values reached in several periods of each exposure day in the real in situ Mediterranean climate environment seem to favour the development of clinker and slag hydration, and therefore, the microstructure development of CEM I and III mortars in the short-term.
- In general, the results of the capillary suction coefficient and the effective porosity obtained in this research would indicate that slag and fly ash cement mortars would have good durability in the short-term for all the studied environments, compared to those prepared using ordinary Portland cement.
- The slag and fly ash cement mortars showed the lowest non-steady state chloride migration coefficients after 90 hardening days, regardless of the exposure environment. This could be due to the higher binding capacity brought by slag and fly ash, as well as to the greater microstructure refinement produced by these additions.
- The microstructure and durability-related results obtained for the studied mortars hardened in the non-optimum laboratory-simulated Mediterranean climate condition, which consisted of exposing the materials to a constant temperature and relative humidity corresponding to the annual average values of both parameters for this climate, were a good a approach to the results observed for those exposed to the real in-situ environment, although there were some differences among them.
- Considering the results obtained in this research, mortars prepared using sustainable cements with slag and fly ash, exposed to both laboratory simulated and real in situ Mediterranean climate environments, showed adequate service properties in the short-term (90 days), and they were similar or even better in comparison to mortars made with ordinary Portland cement without additions.

## Figures and Tables

**Table 1:** Components of the commercial cements used.

Component	CEM I		CEM III		CEM IV	
	UNE-EN 197-1 Standard [37]	Manufacturer Data ^1^	UNE-EN 197-1 Standard [37]	Manufacturer Data ^1^	UNE-EN 197-1 Standard [37]	Manufacturer Data ^1^
Cement	95–100%	95%	20–34%	31%	45–64%	50%
Limestone	-	5%	-	-	-	-
Blast-furnace slag	-	-	66–80%	69%	-	-
Fly ash	-	-	-	-	36–55%	50%

^1^ Specific percentage of each component usually used according to the manufacturer.

**Table 2:** Characteristics of environmental conditions studied

Exposure Condition	Description
Environment A	Optimum laboratory condition (constant 20 °C and 100% RH)
Environment B	Non-optimum laboratory condition representative of the Mediterranean climate (constant 20 °C and 65% RH)
Environment C	Maritime real condition of Mediterranean climate (exposure site at 100 m approximately of Mediterranean Sea)

## References

[1] Demirboğa R. (2007). Thermal conductivity and compressive strength of concrete incorporation with mineral admixtures. Build. Environ..

[2] Ganjian E., Pouya H.S. (2005). Effect of magnesium and sulfate ions on durability of silica fume blended mixes exposed to the seawater tidal zone. Cem. Concr. Res..

[3] Ponikiewski T., Gołaszewski J. (2014). The effect of high-calcium fly ash on selected properties of self-compacting concrete. Arch. Civ. Mech. Eng..

[4] Ortega J.M., Sánchez I., Climent M.A. (2015). Impedance spectroscopy study of the effect of environmental conditions in the microstructure development of OPC and slag cement mortars. Arch. Civ. Mech. Eng..

[5] Glinicki M., Jóźwiak-Niedźwiedzka D., Gibas K., Dąbrowski M. (2016). Influence of Blended Cements with Calcareous Fly Ash on Chloride Ion Migration and Carbonation Resistance of Concrete for Durable Structures. Materials.

[6] Ortega J.M., Esteban M.D., Rodríguez R.R., Pastor J.L., Sánchez I. (2016). Microstructural Effects of Sulphate Attack in Sustainable Grouts for Micropiles. Materials.

[7] Ortega J.M., Esteban M.D., Rodríguez R.R., Pastor J.L., Ibanco F.J., Sánchez I., Climent M.A. (2017). Long-Term Behaviour of Fly Ash and Slag Cement Grouts for Micropiles Exposed to a Sulphate Aggressive Medium. Materials.

[8] Williams M., Ortega J.M., Sánchez I., Cabeza M., Climent M.A. (2017). Non-Destructive Study of the Microstructural Effects of Sodium and Magnesium Sulphate Attack on Mortars Containing Silica Fume Using Impedance Spectroscopy. Appl. Sci..

[9] Ortega J.M., Esteban M.D., Rodríguez R.R., Pastor J.L., Ibanco F.J., Sánchez I., Climent M.A. (2017). Influence of Silica Fume Addition in the Long-Term Performance of Sustainable Cement Grouts for Micropiles Exposed to a Sulphate Aggressive Medium. Materials.

[10] Bijen J. (1996). Benefits of slag and fly ash. Constr. Build. Mater..

[11] Pastor J.L., Ortega J.M., Flor M., López M.P., Sánchez I., Climent M.A. (2016). Microstructure and durability of fly ash cement grouts for micropiles. Constr. Build. Mater..

[12] Ortega J.M., Pastor J.L., Albaladejo A., Sánchez I., Climent M.A. (2014). Durability and compressive strength of blast furnace slag-based cement grout for special geotechnical applications. Mater. Constr..

[13] Climent M.A., Ortega J.M., Sánchez I. (2012). Cement mortars with fly ash and slag—Study of their microstructure and resistance to salt ingress in different environmental conditions. Concrete Repair, Rehabilitation and Retrofitting III, Proceedings of the 3rd International Conference on Concrete Repair, Rehabilitation and Retrofitting (ICCRRR 2012), Cape Town, South Africa, 3–5 September 2012.

[14] Wedding P., Manmohan D., Mehta P. (1981). Influence of Pozzolanic, Slag, and Chemical Admixtures on Pore Size Distribution and Permeability of Hardened Cement Pastes. Cem. Concr. Aggreg..

[15] Geiseler J., Kollo H., Lang E. (1995). Influence of blast furnace cements on durability of concrete structures. ACI Mater. J..

[16] Thomas M.D.A., Scott A., Bremner T., Bilodeau A., Day D. (2008). Performance of slag concrete in marine environment. ACI Mater. J..

[17] Ortega J.M., Albaladejo A., Pastor J.L., Sánchez I., Climent M.A. (2013). Influence of using slag cement on the microstructure and durability related properties of cement grouts for micropiles. Constr. Build. Mater..

[18] Thomas M.D., Matthews J. (2004). Performance of pfa concrete in a marine environment––10-Year results. Cem. Concr. Compos..

[19] Chalee W., Jaturapitakkul C., Chindaprasirt P. (2009). Predicting the chloride penetration of fly ash concrete in seawater. Mar. Struct..

[20] Bouikni A., Swamy R.N., Bali A. (2009). Durability properties of concrete containing 50% and 65% slag. Constr. Build. Mater..

[21] Ortega J.M., Sánchez I., Climent M.A. (2012). Durability related transport properties of OPC and slag cement mortars hardened under different environmental conditions. Constr. Build. Mater..

[22] Scott A.N., Thomas M.D.A., Bremner T.W. Marine performance of concrete containing fly ash and slag. Proceedings of the Canadian Society for Civil Engineering Annual Conference 2009.

[23] Shattaf N.R., Alshamsi A.M., Swamy R.N. (2001). Curing/environment effect on pore structure of blended cement concrete. J. Mater. Civ. Eng..

[24] Pasupathy K., Berndt M., Castel A., Sanjayan J., Pathmanathan R. (2016). Carbonation of a blended slag-fly ash geopolymer concrete in field conditions after 8 years. Constr. Build. Mater..

[25] Polder R.B., De Rooij M.R. (2005). Durability of marine concrete structures—Field investigations and modelling. Heron.

[26] Detwiler R.J., Kjellsen K.O., Gjorv O.E. (1991). Resistance to chloride intrusion of concrete cured at different temperatures. ACI Mater. J..

[27] Çakır Ö., Aköz F. (2008). Effect of curing conditions on the mortars with and without GGBFS. Constr. Build. Mater..

[28] Ramezanianpour A.A., Malhotra V.M. (1995). Effect of curing on the compressive strength, resistance to chloride-*ion* penetration and porosity of concretes incorporating slag, fly ash or silica fume. Cem. Concr. Compos..

[29] Sánchez I., Albertos T.S., Ortega J.M., Climent M.A., Zachar J., Claisse P., Naik T.R., Ganjian E. Influence of environmental conditions on durability properties of fly ash cement mortars. Proceedings of the 2nd International Conference on Sustainable Construction Materials and Technologies.

[30] Ortega J.M., Sánchez I., Climent M.A., Zachar J., Claisse P., Naik T.R., Ganjian E. Influence of environmental conditions on durability of slag cement mortars. Proceedings of the 2nd International Conference on Sustainable Construction Materials and Technologies.

[31] Ortega J.M., Ferrandiz V., Antón C., Climent M.A., Sánchez I., Mammoli A.A., Brebbia C.A. (2009). Influence of curing conditions on the mechanical properties and durability of cement mortars. Materials Characterisation IV: Computational Methods and Experiments.

[32] Sánchez I., Antón C., de Vera G., Ortega J.M., Climent M.A. (2013). Moisture Distribution in Partially Saturated Concrete Studied by Impedance Spectroscopy. J. Nondestruct. Eval..

[33] Ortega J.M., Sánchez I., Antón C., De Vera G., Climent M.A. (2012). Influence of environment on durability of fly ash cement mortars. ACI Mater. J..

[34] Ortega J.M., Sánchez I., Climent M.Á. (2013). Influence of different curing conditions on the pore structure and the early age properties of mortars with fly ash and blast-furnace slag. Mater. Constr..

[35] Chalee W., Ausapanit P., Jaturapitakkul C. (2010). Utilization of fly ash concrete in marine environment for long term design life analysis. Mater. Des..

[36] Ortega J.M., Sánchez I., Cabeza M., Climent M.A. (2017). Short-Term Behavior of Slag Concretes Exposed to a Real In Situ Mediterranean Climate Environment. Materials.

[37] Asociación Española de Normalización y Certificación (AENOR) (2011). UNE-EN 197-1:2000. Cemento. Parte 1: Composición, Especificaciones y Criterios de Conformidad de Los Cementos Comunes.

[38] Asociación Española de Normalización y Certificación (AENOR) (2005). UNE-EN 196-1:2005. Métodos de Ensayo de Cementos. Parte 1: Determinación de Resistencias Mecánicas.

[39] Deutsches Institut für Normung e.V. (1981). Deutsche Norm DIN 50008 Part 1.

[40] Comisión Permanente del Hormigón (2008). Instrucción De Hormigón Estructural EHE-08.

[41] European Committee for Standardization (2004). EN 1992-1-1 Eurocode 2: Design of Concrete Structures-Part1-1: General Rules and Rules for Buildings.

[42] Diamond S. (1999). Aspects of concrete porosity revisited. Cem. Concr. Res..

[43] Diamond S. (2000). Mercury porosimetry. Cem. Concr. Res..

[44] Asociación Española de Normalización y Certificación (AENOR) (2008). UNE 83982:2008. Durabilidad del Hormigón. Métodos de Ensayo. Determinación de La Absorción de Agua Por Capilaridad del Hormigón Endurecido. Método Fagerlund.

[45] Rilem recommendation TC 116-PCD (1999). Permeability of concrete as a criterion of its durability. Mater. Struct..

[46] Nordtest (1999). Mortar and Cement-Based Repair Materials: Chloride Migration Coefficient from Non-Steady-State Migration Experiments (NT Build 492. Concrete).

[47] Wang A., Zhang C., Sun W. (2004). Fly ash effects. Cem. Concr. Res..

[48] Papadakis V.G. (1999). Effect of fly ash on Portland cement systems. Cem. Concr. Res..

[49] Chindaprasirt P., Jaturapitakkul C., Sinsiri T. (2007). Effect of fly ash fineness on microstructure of blended cement paste. Constr. Build. Mater..

[50] Malhotra V.M. (1993). Fly ash, slag, silica fume, and rice-husk ash in concrete. A review. Concr. Int..

[51] Maltais Y., Marchand J. (1997). Influence of curing temperature on cement hydration and mechanical strength development of fly ash mortars. Cem. Concr. Res..

[52] Hanehara S., Tomosawa F., Kobayakawa M., Hwang K. (2001). Effects of water/powder ratio, mixing ratio of fly ash, and curing temperature on pozzolanic reaction of fly ash in cement paste. Cement Concr. Res..

[53] Baroghel-Bouny V. (2007). Water vapour sorption experiments on hardened cementitious materials. Cem. Concr. Res..

[54] Leng F., Feng N., Lu X. (2000). An experimental study on the properties of resistance to diffusion of chloride ions of fly ash and blast furnace slag concrete. Cem. Concr. Res..

